# Protrusive Push versus Enveloping Embrace: Computational Model of Phagocytosis Predicts Key Regulatory Role of Cytoskeletal Membrane Anchors

**DOI:** 10.1371/journal.pcbi.1001068

**Published:** 2011-01-27

**Authors:** Marc Herant, Cheng-Yuk Lee, Micah Dembo, Volkmar Heinrich

**Affiliations:** 1 Department of Biomedical Engineering, Boston University, Boston, Massachusetts, United States of America; 2 Department of Biomedical Engineering, University of California, Davis, Davis, California, United States of America; 3 Department of Chemical Engineering and Materials Science, University of California, Davis, Davis, California, United States of America; University of California San Diego, United States of America

## Abstract

Encounters between human neutrophils and zymosan elicit an initially protrusive cell response that is distinct from the thin lamella embracing antibody-coated targets. Recent experiments have led us to hypothesize that this behavior has its mechanistic roots in the modulation of interactions between membrane and cytoskeleton. To test and refine this hypothesis, we confront our experimental results with predictions of a computer model of leukocyte mechanical behavior, and establish the minimum set of mechanistic variations of this computational framework that reproduces the differences between zymosan and antibody phagocytosis. We confirm that the structural linkages between the cytoskeleton and the membrane patch adherent to a target form the “switchboard” that controls the target specificity of a neutrophil's mechanical response. These linkages are presumably actin-binding protein complexes associating with the cytoplasmic domains of cell-surface receptors that are engaged in adhesion to zymosan and Fc-domains.

## Author Summary

Recent micropipette experiments have provided a unique live view of “one-on-one” interactions between human neutrophils and their phagocytic targets. Our results revealed surprising differences between two prominent immunological pathways: the response to fungal targets (mimicked using zymosan particles), and antibody-mediated phagocytosis. Whereas antibody-coated targets were “pulled” into the cell in a straightforward manner, zymosan particles were internalized only after an initial outward “push”. We hypothesized that structural interactions between the cytoskeleton and the membrane patch adherent to a target play a pivotal role in the control of this target specificity. To verify and refine this hypothesis, we here compare our experimental results with predictions of suitable adaptations of a previously validated computational model of neutrophil mechanical behavior. By optimizing the model to best match our experiments, we corroborate that the primary mechanistic origin of the target-specific cell behavior indeed lies in the strength of cytoskeletal membrane anchors.

## Introduction

**Figure 1:**
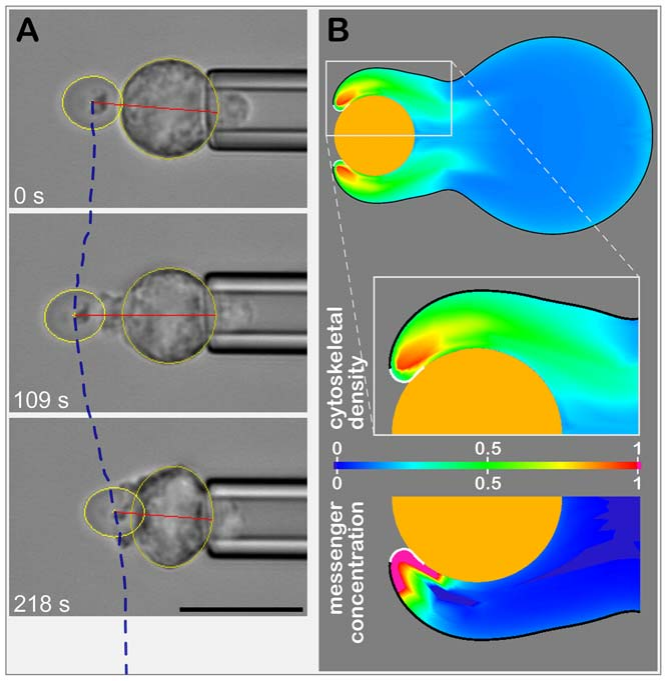
Overview of single-cell/single-target experiment and computer model. (**A**) Example videomicrographs of a phagocytosis experiment showing the typical initial response of a pipette-held neutrophil to adhesive contact with a zymosan particle. The dashed line depicts part of the time-dependent positional trajectory of the target center (measured relative to the opposite edge of the main cell body, red line). The scale bar is 10 µm. (**B**) Snapshot of a computer simulation. The zymosan particle is modeled as a rigid sphere. The insets enlarge the pseudopod engulfing the particle. The leading, white membrane patch marks the region where a signaling messenger is produced. The coloring of the lamella interior reflects the relative density of the cytoskeletal actin network (top half) and the relative concentration of the messenger (bottom half).

Our recent quantitative comparison of the physical responses of human neutrophils to zymosan (an insoluble, particulate fraction from yeast cell walls and prominent model system in the study of fungal infection [1], [2]) and to antibody-coated targets has exposed differences between these two forms of phagocytosis [3]. Zymosan phagocytosis typically commences as a chemotactic-like, pseudopodial protrusion that collides with, and eventually overflows, its target. In contrast, antibody-mediated phagocytosis represents more of an enveloping process in which a thin cellular lamella advances along all sides of a firmly held target to achieve engulfment [3]. The quantitative measure that most clearly illustrates this difference is the distance over which a neutrophil initially pushes an adherent target outwards (Fig. 1A). This push-out distance is 1.03±0.3(SD) µm in the case of zymosan, versus 0.12±0.14(SD) µm for antibody-coated targets. The “detour” in cell deformation at the onset of zymosan phagocytosis proves time-consuming: zymosan engulfment takes ∼2.5 times longer (167±73(SD) s) than the uptake of antibody-coated particles of similar size (66±19(SD) s). The experiments also provided the maximum cortical tension of neutrophils and the fastest speed of target inward motion during the two forms of phagocytosis. A ∼two-fold difference in cortical tension (0.3 mN/m versus 0.14 mN/m, with the higher value observed during zymosan engulfment) contrasts with an indistinguishable target speed (∼33 nm/s) in the two cases. Since the cortical tension is the dominant driver of target inward motion, a conserved target-internalization speed implies that the cytoplasmic viscosity rises concurrently with the cortical tension during phagocytosis, in agreement with a previously reported tight balance between the cortical tension and cytoplasmic viscosity of leukocytes [4].

Such large variations in phagocytic behavior call for a mechanistic explanation. An important clue was provided by our drug-inhibition experiments [3], which exposed a dichotomous mechanical role of actin: in addition to driving the formation of the protrusive pseudopod that pushes zymosan outward at first, actin also participates in the suppression of protrusion during the efficient uptake of antibody-coated targets. We therefore hypothesized that structural interactions between the cytoskeleton and the membrane patch adherent to a target play a pivotal role in the target-specific mechanoregulation of phagocytosis. Although this is a plausible hypothesis, there is no guarantee that it is physically realistic or will explain the observed behavior in detail, be it in terms of cell morphology, target trajectory, or other measurable parameters. It is to verify, adapt, and refine this hypothesis that we present a quantitative analysis of its mechanical implications through the use of a previously validated computational model of neutrophil phagocytosis.

Mechanistic scenarios of target engulfment are “hard-wired” into adaptations of this computational model by translating a set of “cause-effect rules” (or physical/biochemical mechanisms deemed biologically plausible) into mathematical equations, as explained in the Methods section. Our modeling strategy is to implement these equations in a self-consistent manner in a physically realistic computational framework, and solve the resulting problem numerically to predict the cellular behavior. A given model then is iteratively optimized by improving initial guesses of adjustable parameters until the predictions of this framework satisfactorily match experimental observations.

## Methods

### Computer model of autonomous leukocyte deformation

In earlier work, we have developed and extensively validated a finite-element model of the human neutrophil, and applied it successfully to chemotactic and phagocytic mechanics [5], [6]. It is based on the “reactive interpenetrating flow” formalism [5], [7]. Briefly, it conceptualizes the cell interior as a mixture of two materials, the cytoskeleton and the cytosol, enclosed by an envelope, the cell membrane. The cytoskeleton plays the crucial role in controlling the dynamics of cell deformation, whereas the cytosol is a “filler” material whose relocation is driven by pressure gradients. The two phases can convert into each other, reflecting, for instance, the polymerization/depolymerization of G- and F-actin. A movable and deformable boundary endowed with surface tension represents the cortical membrane. Mass and momentum conservation equations determine the evolution of this continuum mechanical model in a self-consistent manner.

To model phagocytic behavior, we have identified a number of necessary prescriptions to account for adhesion, basic signaling, and the generation of mechanical forces [6]. First, we implement an adhesive interaction between the cell surface and the target. Once cell contact with a patch of target is established, detachment is proscribed (as observed). The leading edge of the adherent membrane region is assumed to stimulate transient production of a generic signaling “messenger” that locally triggers conversion of the low-viscosity (cytosolic) phase into the high-viscosity (cytoskeletal) phase, akin to cytoskeletal polymerization (Fig. 1B). The degree of polymerization in turn determines the magnitude of a repulsive (or “disjoining”) force between the membrane and the cytoskeleton that then leads to local protrusion. (This continuum model of protrusion encompasses, but is not limited to, the Brownian ratchet mechanism [8].) Finally, our previous work on neutrophil phagocytosis of antibody-coated beads exposed the necessity to invoke an attractive force between the membrane adherent to the bead and the cytoskeleton that essentially acts to “flatten” the neutrophil onto the bead [6].

Mathematical equations representing the physics of the above mechanistic concepts have been published previously [6]. We solve these model equations numerically through a Galerkin finite-element method using a mesh of quadrilaterals as described in Dembo [9] and Herant et al. [5]. Briefly, the calculation is advanced over a time step Δ*t* (determined by the Courant condition or other fast time scale of the dynamics) by means of five sequential operations:

1. The mesh boundary is advected according to the flow of the cytoskeletal network, and then mesh nodes are repositioned for optimal resolution while preserving mesh topology, boundaries and interfacial surfaces [10].
2. Mass is advected from the old mesh positions to the new mesh using a general Eulerian-Lagrangian scheme with upwind interpolation [11].
3. Diffusive mass transport and chemical reactions are treated according to a backward Euler (implicit) scheme coupled with a Galerkin finite-element treatment of spatial derivatives and boundary conditions.
4. Constitutive laws are used to compute necessary quantities such as viscosities.
5. Finally the momentum equations and the incompressibility condition together with the applicable boundary conditions are discretized using the Galerkin approach, and the resulting system is solved for the pressure, velocity of the cytoskeletal network, and cytosolic velocity.

This computational cycle is repeated until the simulation is complete. Cylindrical symmetry allows the use of a two-dimensional mesh to solve the axisymmetric version of the model equations. Numerical convergence is confirmed by checking that the results are not sensitive to variations of the tolerance of the different iterations performed by the code as well as to variations of the spatial resolution. Calculations are conducted on PC workstations and typically take a few hours per run.

## Results

Two distinct yet closely related questions arise from the experimental comparison of the phagocytosis of zymosan and antibody-coated beads [3]: What are the differences in cell-signaling cascades that are triggered by the different receptors recognizing these targets? What distinguishes the mechanical processes that govern the different forms of particle internalization? Biological phagocytosis research primarily addresses the former (e.g., [12], [13]); in contrast, this paper focuses on the latter, i.e., on physical mechanisms that are fundamental to our understanding of not only phagocytosis but all processes involving eukaryotic cell motility.

We have previously established an optimal computational model describing the phagocytic uptake of antibody-coated beads by human neutrophils [6]. Our strategy here is to take this successful computational model of Fcγ-mediated phagocytosis as a baseline, and determine what changes must be made to recover the behavior observed in the phagocytosis of zymosan. The “virtual” phagocytic target in all simulations is a rigid spherical particle with a diameter of 3.2 µm. We prescribe the time course of the neutrophil cortical tension as measured, i.e., increasing from a resting value of 0.025 mN/m to 0.3 mN/m during the engulfment of zymosan, and to 0.15 mN/m in the case of antibody-coated beads. We previously presented a more elaborate model of the behavior of the cortical tension [14]; however, using observed rather than modeled cortical tension values reduces the complexity of the comparative analysis of phagocytic mechanics that is the primary focus of this paper.

**Figure 2:**
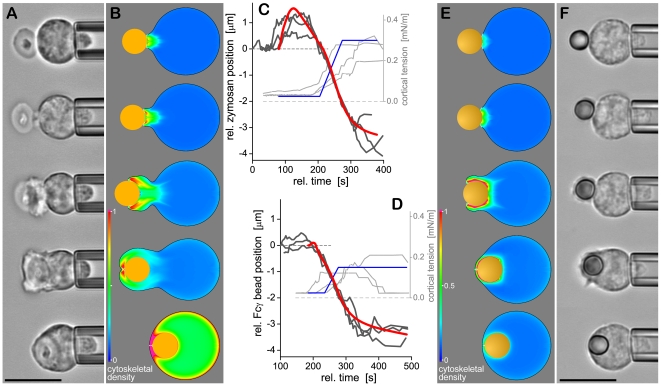
Comparison of optimal computer simulations with experimental observations. (**A and F**) Filmstrips of videomicrographs of neutrophil phagocytosis of a zymosan particle (A) and an antibody-coated bead (F). The scale bar is 10 µm. (**B and E**) Snapshots of our simulations of the engulfment of zymosan (B) and antibody-coated targets (E). Note that the simulations were optimized to reproduce the average experimental behavior and are not the best match to the examples in (A) and (F). The coloring of the cell interior reflects the relative density of the cytoskeletal actin network. (**C and D**) Comparison of time-course graphs of three typical experimental target trajectories (noisy dark-gray curves, cf. Fig. 1A) with the respective results of the optimal simulations (red) for both forms of phagocytosis. Also shown are measured (light-gray curves) as well as prescribed, simplified cortical tension timelines (blue).

Fig. 2 shows the results of our adapted model of zymosan phagocytosis, as well as simulations of Fcγ-mediated phagocytosis using the previously established model [6] (see also Supporting Videos S1 and S2). Differences between the two models are summarized in Table 1. Both simulations are in excellent agreement with corresponding observations. To achieve such agreement in the case of zymosan phagocytosis, the following changes had to be incorporated into our original model of Fcγ-mediated phagocytosis:

- A qualitatively different set of interaction forces at the cell-target interface,
- A lower degree of cytoskeletal polymerization at the pseudopod's leading edge during zymosan engulfment, and
- A significant rise in viscosity within the cell body toward the end of zymosan phagocytosis.

**Table 1:** Summary of mechanoeffectors in the target-specific computer models.

	Phagocytic target	
Mechanical effector	Antibody-coated bead	Zymosan particle
**Adhesion**	upon contact; irreversible	
**Local stimulus of actin polymerization and protrusive force**	100%	75%
**Protrusive force at free membrane**	same	
**Protrusive force at cell-target contact region**	none	50% of above force at free membrane
**Attractive force between adherent membrane and cytoskeleton**	100%	none
**Cortical tension**	linear rise to plateau	
	0.025→0.15 mN/m	0.025→0.3 mN/m
**Cytoplasmic viscosity**	constant throughout	five-fold rise concurrent with tension

We discuss each of these aspects in turn.

**Figure 3:**
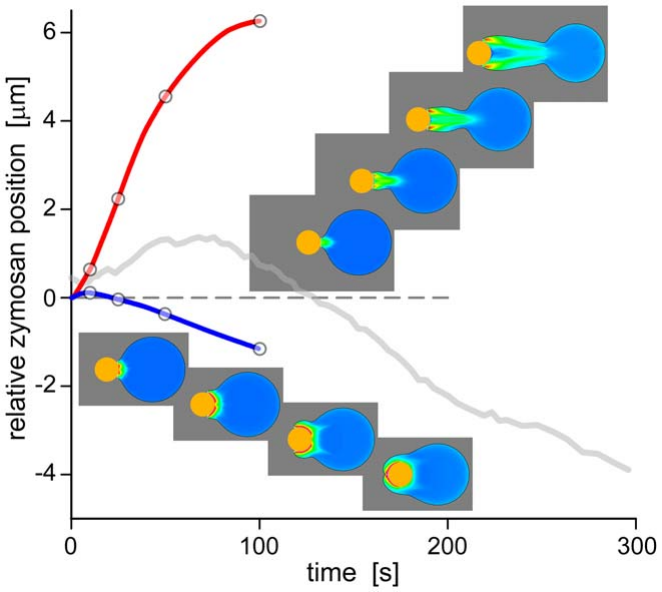
Elimination of potential mechanisms of zymosan phagocytosis due to mismatch between the predictions of the respective computer simulations and experimental results. The noisy, light-gray curve is a “benchmark” positional trace of a zymosan particle as typically measured in single-cell experiments. Trajectories of unsuccessful simulations are shown in blue (original Fcγ model without attractive force) and red (after adding a full-strength protrusive force at the cell-target contact region). Included are snapshots of unsatisfactory simulations corresponding to the time points marked by circles.

We have shown previously that a combination of attractive force and lack of protrusion at the membrane-bead interface accounts for two distinctive features of Fcγ-mediated phagocytosis: the absence of significant outward motion of the bead at the onset of engulfment, and the thin lamellar pseudopod embracing the bead [6]. In contrast, zymosan particles do exhibit significant outward motion before engulfment, and the pseudopods surrounding zymosan are thick (Fig. 2; [3]). Hence we find that the attractive force is superfluous when modeling this case. Yet even after its removal, the modeled outward motion of the target remains much less than typically observed in zymosan phagocytosis (Fig. 3, blue line). To reproduce the measured initial push-out distances of zymosan particles, it is necessary to postulate that in this case, the protrusive force driving out free (non-adherent) membrane also pushes to some extent against the membrane in contact with the target – much as if the contact patch acted as a chemoattractant rather than a locus of contraction-inducing adhesion. On the other hand, when implementing this polymerization-driven protrusion force at the cell-target interface and ascribing to it the same “full” strength as at the free membrane, the resulting outward motion of the target far overshoots the observed distances (Fig. 3, red line). Only by choosing a middle ground—i.e., setting the strength of the protrusive force at the cell-target interface to 50% of the value acting at the free membrane—are we able to recover the correct behavior (Fig. 2).

How does one interpret these differences in local protrusion? Consider a cell-surface patch in adhesive contact with an external rigid object. If the engaged adhesion receptors are strongly anchored in the cytoskeleton, they tightly couple the latter to the external object. Then, any disjoining force (such as due to de novo actin polymerization) must act against the tensile stress in the molecular structures linking the object to the cytoskeleton. A protrusive deformation will ensue only if the disjoining force exceeds the strength of this link. On the other hand, if an external object adheres only via membrane binding without further internal structural linkage, local disjoining forces will drive protrusion as if the contact patch was a region of free membrane. Based on this reasoning, we conclude that the adhesion of human neutrophils to antibody-coated targets exhibits strong cytoskeletal coupling, whereas in phagocytic adhesion to zymosan the cytoskeletal linkage is weak (but not non-existent).

We also see that the completion of engulfment takes longer for zymosan particles than for antibody-coated beads of the same size. This difference in engulfment duration can be accounted for in the simulations of zymosan phagocytosis by a 25% weaker stimulus of polymerization and protrusive force. Finally, the combination of high cortical tension and large initial target-push-out distance should lead to a much faster inward motion of zymosan particles than actually observed. The only remaining parameter that can be adjusted in the simulations to slow down the inward motion of zymosan is the interior viscosity of the cell body. Whereas this viscosity remains constant throughout the simulation of Fcγ-mediated phagocytosis, our model of zymosan phagocytosis implements a five-fold increase of the viscosity that occurs concurrently with the rise of the cortical tension. This change in cytoplasmic viscosity is assumed to take place throughout the cell interior because otherwise, the intracellular region with the lowest viscosity would determine the rate of cell rounding. To match the measurements, this lowest viscosity would have to have the value currently used for the whole cell interior, and the viscosity in the remainder of the cell would be even higher. Physically, the viscosity increase corresponds to a high degree of polymerization and/or cross-linking of cytoskeletal components throughout the cell.

## Discussion

This computational study examines the mechanistic underpinnings of distinct physical responses of human neutrophils to zymosan and antibody-coated targets. A direct quantitative comparison of a finite-element model of the neutrophil with recent single-cell/single-target experiments [3] allows us to corroborate or discard mechanistic hypotheses about the mechanoregulation of phagocytosis. Key to the success of this comparison has been a suitable experimental design, i.e., an essentially axisymmetric configuration that isolated the cell-target interactions of interest from potential interference by other cellular processes (such as cell-substrate interactions of adherently kept immune cells).

Our computational framework integrates the reactive interpenetrating flow formalism [7] with cell adhesion, basic signaling, and the autonomous generation of forces [6]. We use this framework to establish the variations in the modeled interplay of mechanical forces that most closely reproduce the observed differences in cell behavior. This enquiry complements previous studies of zymosan- and antibody-mediated phagocytosis that have focused on differences in receptor-mediated recognition and biochemical signaling [12], [13], [15], [16]. By considering generic mechanistic principles, our computational approach is able to cover mechanical outcomes from underlying processes involving a range of cellular receptors and associated signaling reactions.

Our overall strategy has been to vet mechanistic scenarios of phagocytic target uptake by postulating and testing biologically plausible cause-effect relationships. Additional assumptions implemented in our simulations include the irreversibility of cell-target adhesion and the time dependence of the cortical tension (as measured). We do not impose any particular aspects of the cell morphology; instead, the time courses of both the shape (including surface area) of our “virtual immune cell” as well as the cytoskeletal density distribution (as seen in Figs. 2, B and E, and in the Supporting Videos S1 and S2) are outcomes of the simulation.

Summarizing our findings, a simplified “mechanistic timeline” of zymosan phagocytosis encompasses the following stages:

- Cell-target contact leading to recognition and adhesion.
- Local, transient signaling emanating from the region of fresh contact.
- Local protrusion of the cytoskeleton, resulting in pseudopod formation, gradual growth of the contact region and continual advance of the primary signaling source of actin polymerization.
- Rise of the cortical tension, causing the zymosan particle to be pulled into the cell. Concurrently, the cytoplasmic viscosity increases, reflecting stimulation of the cell as a whole.
- Rounding of the cell and eventual decrease of the cortical tension and cytoplasmic viscosity, completing the uptake of zymosan.

The primary difference between this mechanistic sequence and Fcγ-mediated phagocytosis is the following. Neutrophil contact with an antibody-coated target suppresses cell protrusion directly underneath the cell-target contact region, presumably due to a stronger structural association of the adherent membrane with the cytoskeleton. On a molecular scale, we speculate that these linkages are actin-binding protein complexes that also associate with the cytoplasmic domains of Fcγ-receptors engaged in adhesion. (The postulated difference in cytoskeletal coupling may be due either to distinct strengths of individual linkages between receptors and actin, or to a difference in densities of the engaged receptors.) As a result, protrusion is limited to the cell surface not in contact with the target, leading to a pseudopodial lamella that envelops the target.

Worth highlighting in this physical perspective of phagocytosis is the common role of the cortical tension as primary driver of inward target motion. Note that this contrasts with the notion that an actual inward pulling force, presumably generated by molecular motors, should mainly be responsible for drawing the target into a cell. Neither our experiments (e.g., in the presence of myosin-II inhibitors) [3] nor our modeling work found evidence for a significant participation of such a contractile force in inward target movement. Instead, the cortical-tension-driven tendency of a cell to round up, in conjunction with strong adhesion between the cell membrane and target, appears to be the dominant mechanical cause of target motion into the cell, as also supported by the synchronous onsets of cortical tension rise and target inward movement seen in Figs. 2, C and D.

In closing, this study not only illuminates fundamental mechanisms driving the target-specific physical immune responses of human neutrophils, it also reinforces that the present computational framework represents a biologically plausible and physically realistic model of “virtual immune cells”. In addition to correctly reproducing distinct cell morphologies observed in a range of experiments, this model also matches the dynamics of cell deformation, such as the overall engulfment times, or the time-dependent target trajectories measured in phagocytosis experiments. As an early model for calculation of a spectrum of autonomous cellular motions at this level of complexity, it is a step toward one of the key goals of computational biology, i.e., achieving true predictive power.

## Supporting Information

Video S1 Finite-element computer simulation of phagocytosis of a zymosan particle by a human neutrophil. The zymosan particle is modeled as a rigid sphere (diameter of 3.2 µm, shown in orange). The coloring of the cell interior (legend shown at the left) reflects the relative density of the cytoskeletal actin network. The counter at the upper right gives the elapsed time in seconds.
(1.86 MB MOV)

Video S2 Finite-element computer simulation of phagocytosis of a 3.2 µm (diameter) antibody-coated bead by a human neutrophil. The coloring of the cell interior (legend shown at the left) reflects the relative density of the cytoskeletal actin network. The counter at the upper right gives the elapsed time in seconds.
(1.15 MB MOV)
